# Cardiac Amyloidosis Diagnosis with Magnetic Resonance Imaging: A Case Report

**DOI:** 10.7759/cureus.2330

**Published:** 2018-03-15

**Authors:** Meidi El Issa, Malik El Issa, Besma Sidia

**Affiliations:** 1 Internal Medicine, CHRU Brest; 2 Radiology, Morges' Hospital

**Keywords:** cardiac amyloïdosis, mri, protein, left ventricle, right ventricle, enhancement

## Abstract

Amyloidosis is a rare disease, which can affect various organs, such as the kidneys, heart, liver, respiratory and gastrointestinal tracts, and the nervous system. It still has a bad prognosis nowadays, despite chemotherapy and the new biotherapies. Its physiopathology corresponds to an irreversible, extracellular accumulation of fibrillous proteins in the tissues. Notwithstanding the fact that a clear diagnosis can be made with histology (of solid injured organs or a subcutaneous biopsy), magnetic resonance imaging (MRI) can show various advantages, especially to prove cardiac involvement, with great specificity and sensibility as well. Consequently, the MRI's place can be considered a cornerstone of the diagnosis; more so because biopsies are not routine and easy procedures. Moreover, amyloidosis includes several symptoms, which are sometimes tricky, so the clinician should swiftly consider the usefulness of MRI to get the patient well-oriented and treated.

## Introduction

Cardiac amyloidosis is defined by the clinical manifestation of a process of extracellular accumulation of fibrillar and insoluble amyloid proteins. This self-aggregation results in long-term myocardial hypertrophy. Patients suffering from this pathology then develop heart failure with preserved ejection fraction, conduction disorders, or rhythm disorders. Cardiac amyloidosis is a rare disease whose positive diagnosis is based on immunohistochemical, genetic, hematological, and biochemical tests. Previous studies have demonstrated the value of cardiac magnetic resonance imaging (MRI) for the non-invasive diagnosis of this pathological entity. This case report allows us to illustrate it.

## Case presentation

Here, we report the case of a 69-year-old, obese woman of African descent, with a body mass index of 32. She was followed regularly for multiple myeloma, nephrotic syndrome, and bronchial asthma. Carpal tunnel syndrome was discovered a few months ago based on a symptomatology type of paresthesia. She consulted her cardiologist for a rapidly progressive and disabling New York Heart Association (NYHA) III stage dyspnea associated with lower limb edema. She did not report chest pain, lipothymic discomfort, or syncope but described brief episodes of resting palpitations. Auscultating heart sounds were regular, without murmurs and were well struck. Blood pressure was normally low.

**Figure 1:**
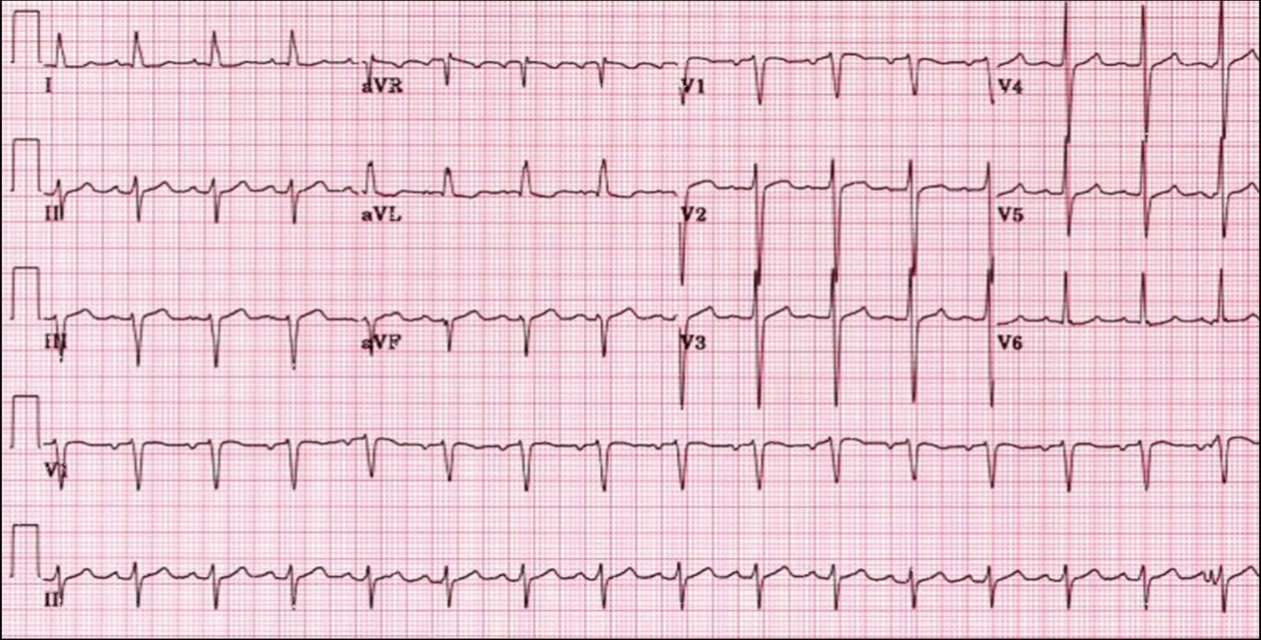
Patient's electrocardiogram (ECG)

The electrocardiogram (ECG) (Figure 1) shows a sinus rhythm, with a left axis due to left anterior hemiblock without disturbance in depolarization.

**Figure 2:**
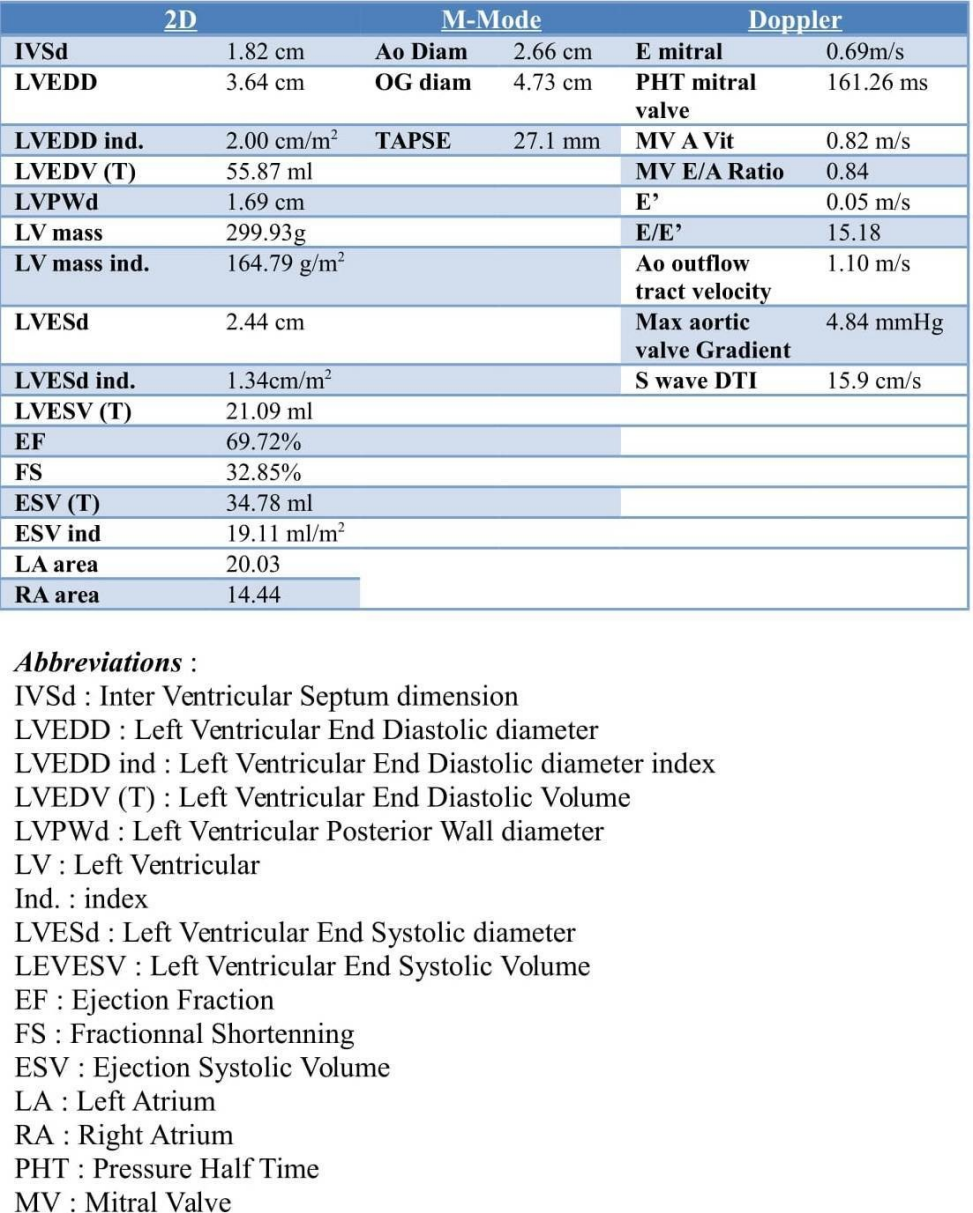
Echocardiography report

The cardiac ultrasound shows undilated ventricles with hypertrophic walls in favor of concentric hypertrophy (Figure 2). The ejection fraction is estimated at 60%-65%. We find a diastolic dysfunction with an increase in filling pressures. The auricular masses are of normal size, uninfiltrated, and there is no valvulopathy. The right ventricle has a normal appearance, with good diastolic function. The pericardium is dry. The myocardium seems to reveal a scintillating appearance suggestive of myocyte infiltration.

A cardiac MRI was required to confirm the hypothesis. The protocol is based on a machine (Siemens Symphony 1.5 Tesla, Munich, Germany) with a dedicated cardiac antenna. The quality of the ECG signal is paramount. Non-synchronized morphological sequences in the three planes are made to define reference planes. Cine steady state free precession (SSFP) sequences in four cavities-longitudinal major axis and minor axis from the base to the apex for the study of the kinetics of the walls, ventricular thickness and mass, ventricular volume, and ejection fraction-are carried out, followed by morphological sequences in short T1 inversion recovery (STIR) in the three planes. Images are collected at first pass after a gadolinium injection followed by Cine SSFP sequences of the flush chambers and the aortic valve, a phase contrast sequence, and a late enhancement sequence in the three planes after calculating the time of optimal inversion during a sequence in Look-Locker (TI Scout, Seimens, Munich, Germany).

The cardiac MRI shows the presence of fat tissue in the subepicardial position with a lipomatous appearance of the interatrial septum. The right atrium is dilated. The right ventricle appears blistered. The overall systolic function of the right ventricle is normal. Contractility is normal, the ejection fraction is 66%, and the left atrium is dilated. The left ventricle is of normal size with concentric hypertrophy predominant on the septal wall. The thickness of the other walls is normal. The apex is free of thrombus. The mitral valve shows a slight thickening of its distal part, with calcification of the posterior ring. The pericardium is dry, not thickened.

In the case of cardiac amyloidosis, a diffuse subendocardial late enhancement affecting the four cavities is classically expected, associated with a pathological kinetics of gadolinium (accumulation of the contrast agent).

**Figure 3:**
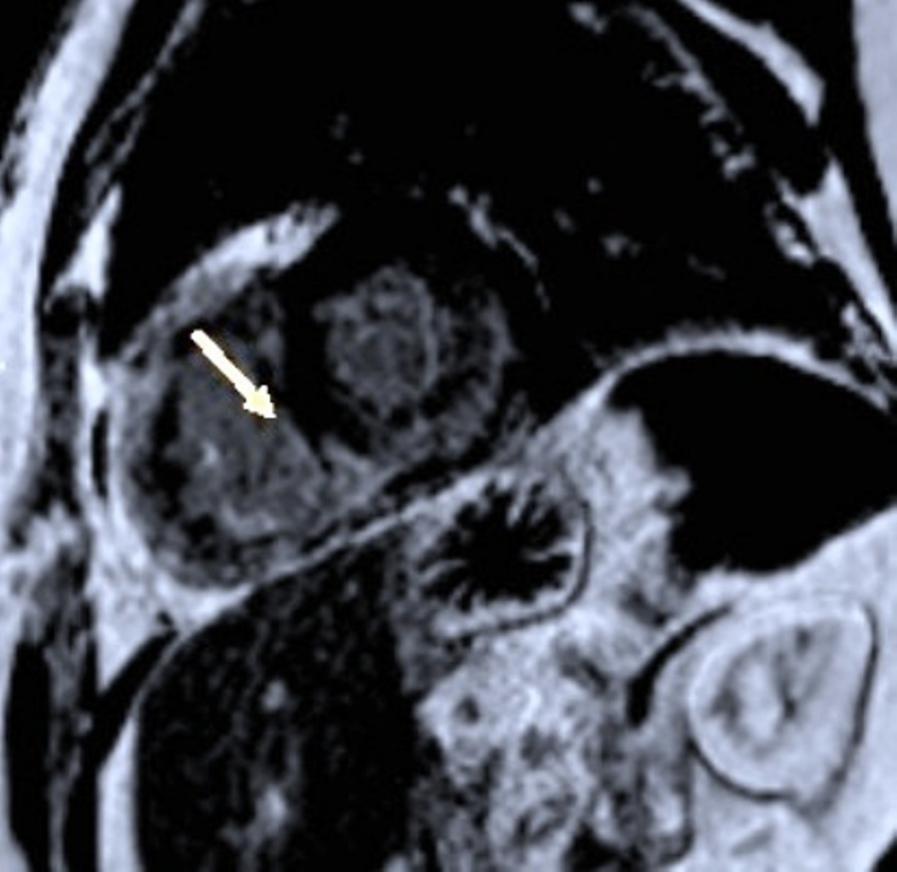
Enhancement of the interventricular septum (SSFP film sequence injected) SSFP: steady state free precession

**Figure 4:**
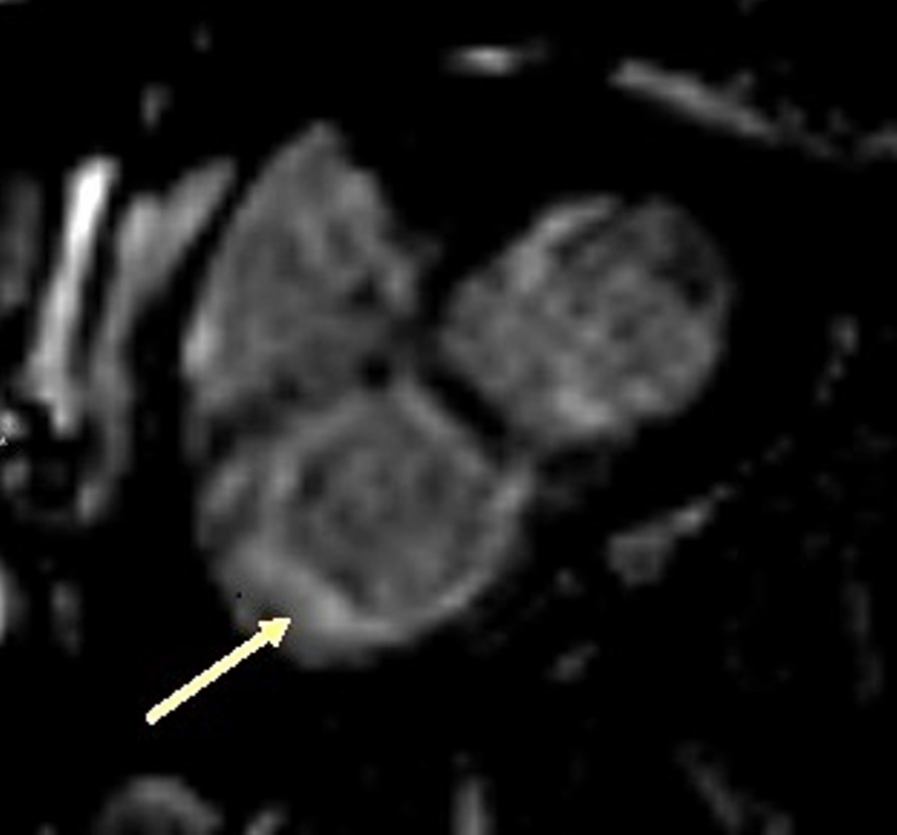
Late subendocardial enhancement at the base of the free wall of the right ventricle (SSFP film) SSFP: steady state free precession

**Figure 5:**
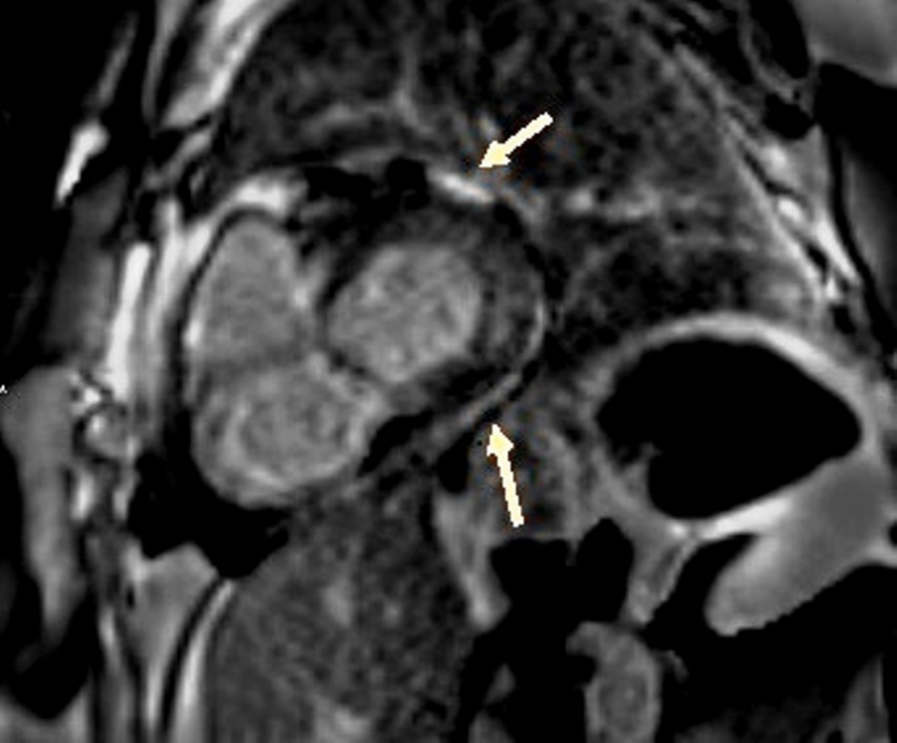
Enhancement of the medio-ventricular anterolateral and infero medial wall of the right ventricle (SSFP film sequence injected) SSFP: steady state free precession

**Figure 6:**
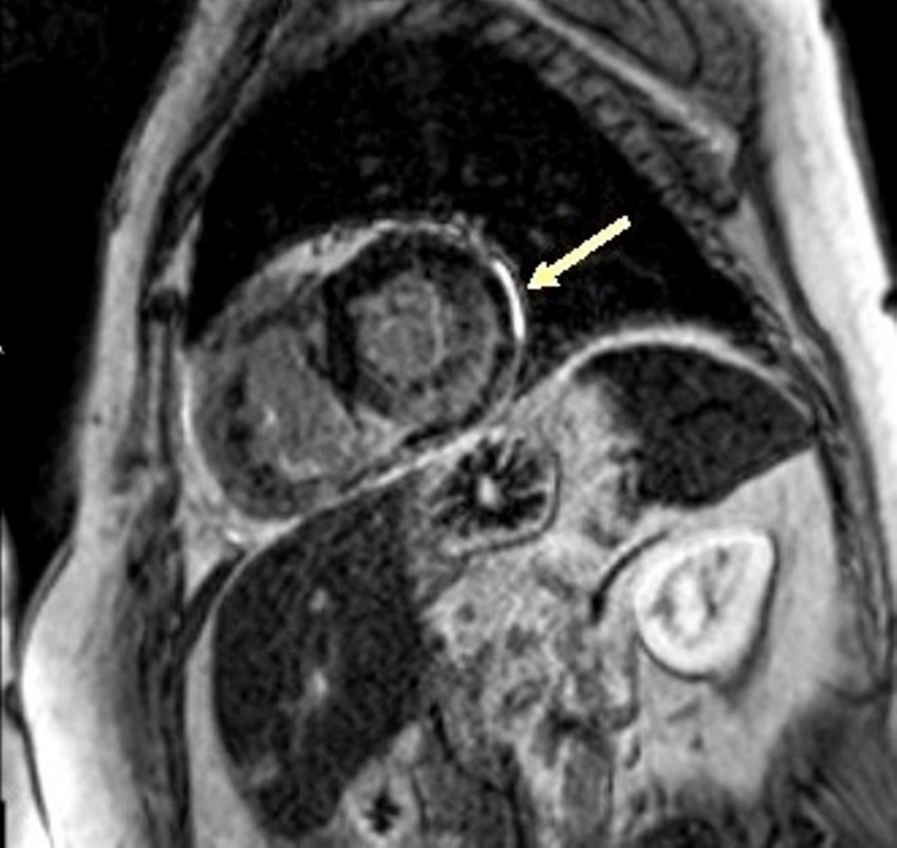
Late enhancement of the left ventricle (sequence in Look-Locker)

In this patient, there was an enhancement of the interventricular septum (Figure 3), a late subendocardial enhancement at the base of the free wall of the right ventricle (Figure 4), on the right side of the interventricular septum, as well as on the medio-ventricular, anterolateral, and inferomedial positions (Figure 5), compatible with cardiac amyloidosis. The inversion time was impossible to measure accurately, and it is well noticed that there is a lateral left ventricular subepicardial late gadolinium enhancement (Figure 6).

## Discussion

Amyloidosis is characterized by the irreversible extracellular insoluble deposits of an amorphous protein complex. The prognosis is often reserved, and the diagnosis is based on the discovery of amyloid deposits compressing the cardiomyocytes, appearing as a green birefringence in polarized light after staining of the Congo red biopsied tissue.

Three forms (amyloidosis light-chain (AL), transthyretin-related amyloidosis (TTRA), and amyloid A (AA)) of amyloidosis are known, depending on the fibrillar protein involved.

The first form, AL monoclonal immunoglobulin chains (primary amyloidosis), represents 50% of cases with cardiac involvement and frequent cardiac failure. AL amyloidosis is mainly related to monoclonal gammapathies of undetermined significance (MGUS) or myeloma. These gammapathies are rarely complicated by amyloidosis but account for more than 60% of amyloid heart disease.

The second form, TTRA, can also affect the myocardium. Transmission is autosomal dominant. Tissue involvement varies according to the mutation. The TTRA Val30Met mutation is the most common and occurs in patients of Portuguese origin with neuropathy at the age of 25 to 30 years. Some mutations (Val122Ile, Ser77Tyr) affect mainly or exclusively the heart. These cases of amyloidosis are underestimated in the population of cardiological patients. Val122Ile mutation discovery in patients of African origin is becoming more common.

The last form, AA, is defined by the presence of an amyloid A protein in the plasma, occurring in the context of chronic inflammatory systemic diseases (secondary amyloidosis).

The involvement of the autonomic nervous system can be at the forefront (AL form and hereditary TTRA form) and affect all the autonomic functions, leading to gastroparesis, which is responsible for uncontrollable vomiting, sources of dyskalemia, disorders of the genitourinary functions, sources of infection, and severe orthostatic hypotension. The cardiac manifestations are related to myocardial infiltration giving rise to either conduction disorders (atrioventricular block) or hyperexcitability (atrial fibrillation, flutter). Outbreaks of heart failure are also common.

The extra-cardiac manifestations vary according to the type of amyloidosis, the neurological disorders concern mainly the autonomic nervous system, and the peripheral nerves (impaired length-dependent fibers). Clinically, peripheral nerve damage is related to sensory disturbances associated with paresthesia of the extremities. It is not uncommon to see diagnostic errors in the elderly; the neurological symptoms are then wrongly attributed to other neurological pathologies (diabetic neuropathy, spondylolisthesis). The electromyogram is the gold standard test that can be used to detect the involvement of small fibers, to quantify and monitor the clinical course.

The classic cardiac presentation corresponds to imaging with a rather concentric and asymmetric myocardial hypertrophy contrasting with electrocardiographic microvoltage. The systolic function may be normal at an early stage and impaired after the disease is advanced, with a restrictive transmural profile. The filling pressures are, in fact, most of the time increased to the advanced stage. Wall atrial thrombi are possible. Pericardial pleural effusions are frequently observed (triad: right ventricular hypertrophy, dilated left atrium, and pericardial effusion).

In a cardiac MRI, primary and secondary amyloidosis were well described by Van Geluwe in 2006 [1] and Hansen in 2007 [2]. Nodular thickening (> 6 mm) of the free wall of the right atrium and atrial septum can be observed [3]. The phenomenon of late enhancement after gadolinium injection has been studied in several small series [4-5] as well as in larger ones [6]. According to Perugini, in 2006 [7], the late enhancement after gadolinium is found in 76% of patients with systemic amyloidosis with positive elements in cardiac sonography: septal thickness > 12 mm, homogeneous thickening of the atrioventricular valves, septal thickening, hyperechoic appearance of the myocardium, and/or interventricular septum (shiny aspect or granite). The mid-ventricular regions are the most frequently affected. A late diffuse subendocardial enhancement was observed in 69% of patients with amyloidosis by Pennell in 2005 [8], corresponding to a significantly lower T1 in this territory. A characteristic distribution is represented by the "zebra" appearance with a subendocardial layer (almost circumferential) and an epicardial hyperintense line separated by a medio-paretinal hypointense zone. The other myocardial walls (right ventricle, atrium) also appear hyperintense.

The presence of a hyper-signal has a significant sensitivity to gauge cardiac involvement during amyloidosis. One of the most specific signs is the impossibility or great difficulty in correctly adjusting the inversion time to discriminate the myocardial blood pool from the walls because of the accumulation of gadolinium in insoluble fibrillar proteins (of significant interest in the sequences, say in the phase sensitive inversion recovery (PSIR) phase). The Look-Locker (TI scout) sequences have a strong diagnostic value because they show a hypointense signal of the myocardium for TI values shorter than the blood pool. For Krombach in 2007 [9], the basic measure of myocardial T1 (without gadolinium) also makes it possible to identify the interstitial infiltration of amyloid deposits because T1 will be significantly increased. In a series of 33 patients [10], 15 with proven cardiac amyloidosis, the cardiac MRI showed a sensitivity of 80% and a specificity of 94% in the diagnosis.

MRI thus confirms the diagnosis of cardiac amyloidosis based on the presence of a lowered circulating/subendocardial blood gradient and an increased subendocardial/subepicardial gradient.

Practical implications

The MRI definitely represents a real alternative to biopsy (especially myocardial). This examination has a significant specificity and sensitivity for the diagnosis of amyloidosis, which gives it a place of choice in the diagnostic approach, in addition to echocardiography.

Access to cardiac MRI, however, remains limited in many test centers and requires good experience in image interpretation to confirm the diagnosis of amyloidosis.

## Conclusions

Indeed, this exam leads to a detailed analysis of cardiac function and the extent of any involvement, including the existence of atrial thrombus. In addition, the reliability of the different protocols used is a real advantage: sequences and contrast agents. PSIR sequences, TI Look-Locker, and enhancement sequences are crucial. However, there is still a lack of data to improve screening and make diagnosis more accurate. The gold standard remains the endomyocardial biopsy with histology and staining with Congo red, showing extracellular infiltration by a fibrillar protein, predominant in the subendocardial, with fibrosis possibly associated. In case of systemic amyloidosis, less invasive biopsies can be performed (rectal, subcutaneous abdominal fat, and salivary glands).
